# Mortality and Recurrence Risk in Relation to the Use of Lipid-Lowering Drugs in a Prospective Breast Cancer Patient Cohort

**DOI:** 10.1371/journal.pone.0075088

**Published:** 2013-09-25

**Authors:** Stefan Nickels, Alina Vrieling, Petra Seibold, Judith Heinz, Nadia Obi, Dieter Flesch-Janys, Jenny Chang-Claude

**Affiliations:** 1 Division of Cancer Epidemiology, German Cancer Research Center (DKFZ), Heidelberg, Germany; 2 Department for Health Evidence, Radboud University Medical Centre, Nijmegen, The Netherlands; 3 Department of Cancer Epidemiology/Clinical Cancer Registry, University Medical Center Hamburg-Eppendorf, Hamburg, Germany; 4 Department of Medical Biometrics and Epidemiology, University Medical Center Hamburg-Eppendorf, Hamburg, Germany; The Norwegian University of Science and Technology (NTNU), Norway

## Abstract

Lipid-lowering drugs are used for the prevention of cardiovascular diseases. Statins are the most commonly used lipid-lowering drugs. Evidence from preclinical and observational studies suggests that statins might improve the prognosis of breast cancer patients. We analyzed data from the German MARIEplus study, a large prospective population-based cohort of patients aged 50 and older, who were diagnosed with breast cancer between 2001 and 2005. For overall mortality, breast-cancer specific mortality, and non-breast-cancer mortality, we included 3189 patients with invasive breast cancer stage I–IV, and for recurrence risk 3024 patients with breast cancer stage I–III. We used Cox proportional hazards models to assess the association with self-reported lipid-lowering drug use at recruitment. We stratified by study region, tumor grade, and estrogen/progesterone receptor status, and adjusted for age, tumor size, nodal status, metastases (stage I–IV only), menopausal hormone treatment, mode of detection, radiotherapy, and smoking. Mortality analyses were additionally adjusted for cardiovascular disease, diabetes mellitus and body-mass index. During a median follow-up of 5.3 years, 404 of 3189 stage I–IV patients died, and 286 deaths were attributed to breast cancer. Self-reported use of lipid-lowering drugs was non-significantly associated with increased non-breast cancer mortality (Hazard ratio (HR) 1.49, 95% confidence interval (CI) 0.88–2.52) and increased overall mortality (HR 1.21, 95% CI 0.87–1.69) whereas no association with breast cancer-specific mortality was found (HR 1.04, 0.67–1.60). Restricted to stage I–III breast cancer patients, 387 recurrences occurred during a median follow-up of 5.4 years. We found lipid-lowering drug use to be non-significantly associated with a reduced risk of recurrence (HR 0.83, 95% CI 0.54–1.24) and of breast cancer-specific mortality (HR 0.89, 95% CI 0.52–1.49). Although compatible with previous findings of an improved prognosis associated with statin use, our results do not provide clear supportive evidence for an association with lipid-lowering drug use due to imprecise estimates.

## Introduction

Lipid-lowering drugs are used for the prevention of cardiovascular diseases. Statins are the most frequently prescribed lipid-lowering drugs, accounting for 89% of all lipid-lowering drug prescriptions in Germany [1]. Primarily, statins inhibit HMG-CoA reductase, the key enzyme of intracellular cholesterol synthesis, resulting in decreasing blood lipid levels. Evidence from preclinical research indicates that statins might have anticancerogenic properties by inducing apoptosis and by reducing tumor growth, angiogenesis, and metastasis [2]–[5]. Observational studies consistently show no evidence for an influence of statins on breast cancer risk [6]. Thus far, four studies reported on the effects of statin intake on the prognosis of breast cancer [7]–[10]: One recent study reported a reduced breast-cancer-specific mortality. Two studies showed a statistically significant reduced risk of breast recurrence [7], [8], and one study reported a non-significant reduced risk [9]. Here, we assessed the effect of self-reported use of lipid-lowering drugs at recruitment on risk of recurrence as well as of mortality in a large cohort of breast cancer patients of the German MARIEplus study.

### Patients and Methods

#### Ethics statement

The study was approved by the ethics committees of the University of Heidelberg and of the Hamburg Medical Council, and the Medical Board of the State of Rheinland-Pfalz. It was conducted in accordance with the Declaration of Helsinki, and all study participants gave written informed consent.

#### Study population

Our study population consisted of participants of the MARIEplus study, a cohort of breast cancer patients recruited from the MARIE study, a population-based case-control study of breast cancer risk [11]. The cohort consisted of patients aged between 50 and 74 years at diagnosis, who were diagnosed with histologically confirmed in situ or invasive breast cancer. Patients were diagnosed with breast cancer between January 2001 and September 2005 in the region of Hamburg, and between August 2002 and July 2005 in the Rhein-Neckar-Karlsruhe region.

A total of 3813 patients participated and provided relevant data. After excluding patients with in-situ-tumors (n = 231) or previous other tumors (n = 231), those with missing information on tumor stage (n = 2), previous tumors (n = 10) or lipid-lowering drug intake (n = 3) and those who had received neo-adjuvant chemotherapy (n = 147), 3189 patients were available for mortality analyses. For analysis of recurrence, we further excluded patients diagnosed with tumor stage IV (n = 88) and patients with missing information on recurrence (n = 77), resulting in 3024 patients with breast cancer stages I – III available for analysis. To assess our results in a more homogeneous group, we repeated all analyses restricted to postmenopausal women diagnosed with breast cancer stages I – III (n = 2755) as sensitivity analysis.

#### Data collection

Clinical and pathological characteristics of the tumors were abstracted from hospital and pathology records. All patients were interviewed at recruitment (2002–2005) by trained personnel to obtain information on socio-demographic factors, anthropometric measures, medication, menopausal hormone treatment (MHT) exposure, and other established and potential risk factors for breast cancer. Regarding the use of medication, patients were asked if they ever took lipid-lowering drugs on a regular basis, i.e. at least for one year. If yes, patients were asked if they were still taking lipid-lowering drugs. We dichotomized the information as current exposure to lipid-lowering drugs at time of recruitment and never/past exposure as reference category.

#### Outcome assessment

Vital status of participants was determined through population registries up to the end of 2009 (100% completeness vital status follow-up), and all deaths were verified by death certificates. Information on recurrences or second cancers was collected via telephone interviews conducted between May and September 2009. In addition, information was extracted from clinical records or requested from treating physicians to verify self-reported events (>90% of self-reported events verified) or to obtain corresponding information on deceased patients or patients who did not participate in the interview (98% completeness of recurrence follow-up). Participant information was censored at date of the event of interest, last contact or 31 December 2009, whichever came first.

#### Statistical analyses

We used Cox proportional-hazards models to analyze the association of self-reported intake of lipid-lowering drugs at recruitment with overall mortality, breast cancer-specific mortality, mortality from causes other than breast cancer, and recurrence (ipsilateral/contralateral/local/regional invasive recurrence, distant recurrence). To account for competing risks, deaths other than the respective event of interest have been censored in the analysis of the outcomes breast-cancer mortality, mortality from other causes, and recurrence. We applied left truncation to account for possible survival bias due to a time lag between diagnosis and interview of patients [12].

All models are stratified by study region and adjusted for age at recruitment (continuous). For the adjusted models, we included the following prognostic variables based on prior knowledge: Tumor size (< = 2 cm, >2–< = 5 cm, >5 cm, growth into chest wall/skin), nodal status (number of affected lymph nodes: 0, 1–3, 4–9, > = 10), metastases (dichotomized, included only in mortality analyses of stage I–IV patients). Checking the proportional hazards assumption resulted in strong evidence for a time-dependent effect of grade (low, moderate, high) and estrogen/progesterone receptor status (ER+PR+, ER+PR−/ER−PR+, ER−PR−), we therefore stratified the analyses by grade and receptor status [13]. In the analyses of the mortality outcomes, we additionally included the covariates cardiovascular disease, diabetes mellitus and BMI, because they are indications for the prescription of lipid-lowering drugs and therefore strongly related to both exposure and outcome. Additional potential confounding variables for which established prior knowledge is limited or lacking were evaluated in the overall mortality analyses via backward elimination and retained in all models if p<0.05. The following four variables were included: menopausal hormone treatment (MHT) at diagnosis (yes, no), mode of detection (self-discovered, by physician), radiotherapy (yes, no), smoking status (never, former, current). The following variables were not retained: HER2 status (HER2+, HER2−), type of surgery (ablatio, breast conserving), chemotherapy (yes, no), alcohol consumption (no alcohol consumption, <19 g/day, > = 19 g/day), body mass index (BMI) (18.5–<25 kg/m^2^, <18.5 kg/m^2^, 25–<30 kg/m^2^, > = 30 kg/m^2^), diabetes mellitus (yes, no), cardiovascular disease (yes, no), occupational status (low, medium, high), formal education (low, medium, high), leisure time physical activity since age 50 (<28 metabolic equivalent hours (METh) per week, > = 28 METh/week).

Analyses were performed using the procedures PROC FREQ, PROC MEANS, and PROC PHREG of SAS 9.2 (SAS Institute, Cary, NC, USA), and all statistical tests were two-sided (a = 0.05).

## Results

We included 3189 stage I–IV breast cancer patients in the mortality analyses and 3024 stage I–III patients in the recurrence analyses. Table 1 and Table 2 show the characteristics of both groups according to lipid-lowering drug use. The prevalence of lipid-lowering drug use is nearly ten percent. Lipid-lowering drug users tended to be older than non-users and had a higher prevalence of cardiovascular disease and diabetes mellitus. The proportion of postmenopausal women and overweight women was somewhat higher, but the prevalence of MHT was lower. Lipid-lowering drug users had a lower occupational status and a lower formal education. Tumor characteristics were similar in the two groups, with the exception of nodal status. The proportion of tumors with affected lymph nodes was lower among lipid-lowering drug users. Lipid-lowering drug users were less likely to have received chemotherapy.

**Table 1:** Baseline demographic and health-related behavior characteristics of breast cancer patients by lipid-lowering drug use at recruitment.

Characteristics		stage I–IV (n = 3189)		stage I–III (n = 3024)	
		lipid-loweringdrug use	no lipid-loweringdrug use	lipid-loweringdrug use	no lipid-loweringdrug use
Patients, n (%)		305 (9.6)	2884 (90.4)	287 (9.5)	2737 (90.5)
Study region	Hamburg	166 (9.4)	1610 (90.7)	156 (9.3)	1517 (90.7)
	Rhein-Neckar-Karlsruhe	139 (9.8)	1274 (90.2)	131 (9.7)	1220 (90.3)
Age at diagnosis, years	50–54	17 (5.6)	431 (14.9)	16 (5.6)	416 (15.2)
	55–59	33 (10.8)	654 (22.7)	30 (10.5)	614 (22.4)
	60–64	85 (27.9)	839 (29.1)	81 (28.2)	805 (29.4)
	65–69	113 (37.1)	670 (23.2)	107 (37.3)	631 (23.1)
	> = 70	57 (18.7)	290 (10.1)	53 (18.5)	271 (9.9)
menopausal status	peri	10 (3.3)	269 (9.3)	10 (3.5)	259 (9.5)
	post	295 (96.7)	2615 (90.7)	277 (96.5)	2478 (90.5)
Menopausal hormonetreatment at recruitment	No	186 (61.0)	1506 (52.2)	173 (60.3)	1416 (51.7)
	Yes	116 (38.0)	1357 (47.1)	112 (39.0)	1302 (47.6)
	Missing	3 (1.0)	21 (0.7)	2 (0.7)	19 (0.7)
BMI	18.5–<25 kg/m2	217 (71.2)	2133 (74.0)	208 (72.5)	2040 (74.5)
	<18.5 kg/m2	7 (2.3)	84 (2.9)	7 (2.4)	83 (3.0)
	25–<30 kg/m2	72 (23.6)	574 (19.9)	65 (22.7)	531 (19.40)
	> = 30 kg/m2	9 (3.0)	93 (3.2)	7 (2.4)	83 (3.03)
Smoking status	Never	168 (55.1)	1506 (52.2)	157 (54.7)	1427 (52.1)
	Former	85 (27.9)	793 (27.5)	82 (28.6)	765 (28.0)
	Current	52 (17.1)	585 (20.3)	48 (16.7)	545 (19.9)
Alcohol consumption	No alcohol consumption	50 (16.4)	406 (14.1)	48 (16.7)	373 (13.6)
	<19 g/day	220 (72.1)	2082 (72.2)	206 (71.8)	1985 (72.5)
	> = 19 g/day	34 (11.2)	392 (13.6)	32 (11.2)	375 (13.7)
	Missing	1 (0.3)	4 (0.1)	1 (0.4)	4 (0.2)
Diabetes mellitus	No	235 (77.1)	2671 (92.6)	225 (78.4)	2534 (92.6)
	Yes	69 (22.6)	208 (7.2)	61 (21.3)	200 (7.3)
	Missing	1 (0.3)	5 (0.2)	1 (0.4)	3 (0.1)
Cardiovascular disease	No	78 (25.6)	1512 (52.4)	76 (26.5)	1450 (53.0)
	Yes	227 (74.4)	1372 (47.6)	211 (73.5)	1287 (47.0)
Occupation	Low	131 (43.0)	1022 (35.4)	122 (42.5)	964 (35.2)
	Medium	122 (40.0)	1121 (38.9)	116 (40.4)	1056 (38.6)
	High	50 (16.4)	729 (25.3)	47 (16.4)	706 (25.8)
	Missing	2 (0.7)	12 (0.4)	2 (0.7)	11 (0.4)
Education	Low	213 (69.8)	1644 (57.0)	201 (70.0)	1549 (56.6)
	Medium	59 (19.3)	806 (28.0)	53 (18.5)	771 (28.2)
	High	33 (10.8)	433 (15.0)	33 (11.5)	416 (15.2)
	Missing	0	1 (0.1)	0	1 (0.1)
Leisure time physical activitysince age 50	<28 METh/week	79 (25.9)	779 (27.0)	73 (25.4)	737 (26.9)
	> = 28 METh/week	217 (71.2)	2070 (71.8)	205 (71.4)	1968 (71.9)
	Missing	9 (3.0)	35 (1.2)	9 (3.1)	32 (1.2)

**Table 2:** Baseline tumor and treatment characteristics of breast cancer patients by lipid-lowering drug use at recruitment.

Characteristics		stage I–IV (n = 3189)		stage I–III (n = 3024)	
		lipid-loweringdrug use	no lipid-loweringdrug use	lipid-loweringdrug use	no lipid-loweringdrug use
Patients, n (%)		305 (9.6)	2884 (90.4)	287 (9.5)	2737 (90.5)
Stage	I	140 (45.9)	1311 (45.5)	138 (48.1)	1275 (46.6)
	II	121 (39.7)	1181 (41.0)	117 (40.8)	1149 (42.0)
	III	32 (10.5)	316 (11.0)	32 (11.2)	313 (11.4)
	IV	12 (3.9)	76 (2.6)	0	0
Histological grade	Low	53 (17.4)	574 (19.9)	50 (17.4)	548 (20.0)
	Moderate	166 (54.4)	1526 (52.9)	158 (55.1)	1451 (53.0)
	High	84 (27.5)	771 (26.7)	77 (26.8)	725 (26.5)
	Missing	2 (0.7)	13 (0.5)	2 (0.7)	13 (0.5)
Tumor size	< = 2 cm	167 (54.8)	1655 (57.4)	161 (56.1)	1602 (58.5)
	>2–< = 5 cm	115 (37.7)	1041 (36.1)	105 (36.6)	979 (35.8)
	>5 cm	14 (4.6)	105 (3.6)	14 (4.9)	92 (3.4)
	Growth into chest wall/skin	9 (3.0)	78 (2.7)	7 (2.4)	61 (2.2)
	Missing	0	5 (0.2)	0	3 (0.1)
Nodal status, affectedlymph nodes	0	216 (70.8)	1904 (66.0)	209 (72.8)	1834 (67.0)
	1–3	59 (19.3)	707 (24.5)	54 (18.8)	668 (24.4)
	4–9	21 (6.9)	162 (5.6)	18 (6.3)	149 (5.4)
	> = 10	9 (3.0)	107 (3.7)	6 (2.1)	86 (3.2)
	Missing	0 (0.0)	4 (0.1)	0	0
Metastases at recruitment	No metastases	293 (96.1)	2808 (97.4)	287 (100.0)	2737 (100.0)
	Metastases	12 (3.9)	76 (2.6)	0	0
ER/PR status	ER+PR+	193 (63.3)	1857 (64.4)	182 (63.4)	1765 (64.5)
	ER+PR−/ER−PR+	61 (20.0)	539 (18.7)	57 (19.9)	505 (18.5)
	ER−PR−	50 (16.4)	488 (16.9)	47 (16.4)	467 (17.1)
	Missing	1 (0.3)	0	1 (0.4)	0
HER2 status	HER2+	54 (17.7)	520 (18.0)	48 (16.7)	489 (17.9)
	HER2−	219 (71.8)	2099 (72.8)	207 (72.1)	1991 (72.7)
	Missing	32 (10.5)	265 (9.2)	32 (11.2)	257 (9.4)
Type of surgery	Ablatio	81 (26.6)	850 (29.5)	75 (26.1)	776 (28.4)
	Breast conserving	222 (72.8)	2018 (70.0)	210 (73.2)	1946 (71.1)
	Missing	2 (0.7)	16 (0.6)	2 (0.7)	15 (0.6)
Chemotherapy	No	173 (56.7)	1377 (47.8)	163 (56.8)	1310 (47.9)
	Yes	127 (41.6)	1457 (50.5)	121 (42.2)	1386 (50.6)
	Missing	5 (1.6)	50 (1.7)	3 (1.1)	41 (1.5)
Radiotherapy	No	54 (17.7)	572 (19.8)	44 (15.3)	499 (18.2)
	Yes	245 (80.3)	2276 (78.9)	237 (82.6)	2207 (80.6)
	Missing	6 (2.0)	36 (1.3)	6 (2.1)	31 (1.1)
Endocrine therapy	No tamoxifen and aromatase inhibitor use	54 (17.7)	454 (15.7)	53 (18.5)	442 (16.2)
	Ever tamoxifen or aromatase inhibitor use	239 (78.4)	2270 (78.7)	225 (78.4)	2150 (78.6)
	Missing	12 (3.9)	160 (5.6)	9 (3.1)	145 (5.3)
Mode of detection	self-discovered (palpation, secretion, doctor visitbecause of pain)	161 (52.8)	1572 (54.5)	149 (51.9)	1477 (54.0)
	discovered by routine investigation, mammography, ultrasound	142 (46.6)	1303 (45.2)	136 (47.4)	1252 (45.7)
	Missing	2 (0.7)	9 (0.3)	2 (0.7)	8 (0.3)

During a median follow-up of 5.3 years, 404 of 3189 stage I–IV patients died, and 286 deaths were attributed to breast cancer (Table 3). The proportion of deaths among lipid-lowering drug users was higher than among non-users (16.1% vs. 12.3%), and the difference was more apparent for non-breast cancer mortality (6.6% vs. 3.4%) than for breast cancer-specific mortality (9.5% vs. 8.9%). Among 3024 stage I–III patients, 387 recurrences occurred during a median follow-up of 5.4 years, with only a small difference between lipid-lowering drug users and non-users (11.9% vs. 12.9%).

**Table 3:** Follow-up time and events of breast cancer patients by lipid-lowering drug use at recruitment.

Outcomes assessed	stage I–IV (n = 3189)		stage I–III (n = 3024)	
	lipid-loweringdrug use	no lipid-loweringdrug use	lipid-loweringdrug use	no lipid-loweringdrug use
n patients (%)	305 (9.6)	2884 (90.4)	287 (9.5)	2737 (90.5)
Overall mortality: n events (%)	49 (16.1)	355 (12.3)	39 (13.6)	298 (10.9)
Breast cancer-specific mortality: n events (%)	29 (9.5)	257 (8.9)	20 (7.0)	201 (7.3)
Non-breast cancer mortality: n events (%)	20 (6.6)	98 (3.4)	19 (6.6)	97 (3.5)
Follow-up time for mortality: person years (median)	1552 (5.1)	15411 (5.3)	1491 (5.2)	14748 (5.4)
Recurrence: n events (%)	–	–	34 (11.9)	353 (12.9)
Follow-up time for recurrence: person years (median)	–	–	1412 (5.2)	14028 (5.5)

Use of lipid-lowering drugs was associated with not statistically significant increased non-breast cancer mortality (HR 1.49, 95% CI 0.88–2.52) as well as overall mortality (HR 1.21, 95% CI 0.87–1.69) in adjusted models (Table 4). There was no association with breast cancer-specific mortality (HR 1.04, 95% CI 0.67–1.60). When restricting the analyses to stage I–III patients, the associations of overall mortality and non-breast cancer mortality were essentially unchanged (HR 1.14, 95% CI 0.78–1.66; HR 1.44, 95% CI 0.84–2.46). Use of lipid-lowering drugs was associated with a non-significantly reduced risk of recurrence (HR 0.83, 95% CI 0.54–1.24) as well as of breast-cancer specific mortality (HR 0.89, 95% CI 0.52–1.49). Restricting the analyses to postmenopausal patients did not alter the estimates substantially.

**Table 4:** Hazard ratios for mortality and recurrence associated with lipid-lowering drug use at recruitment, compared to past or never use.

		crude*		adjusted**	
Patients	Outcome	n	HR (95% CI)	n	HR (95% CI)
stage I–IV	Overall mortality	3189	1.25 (0.93–1.70)	3085	1.21 (0.87–1.69)
	Breast cancer-specific mortality	3189	1.08 (0.73–1.59)	3085	1.04 (0.67–1.60)
	Non-breast cancer mortality	3189	1.66 (1.02–2.69)	3085	1.49 (0.88–2.52)
stage I–III	Overall mortality	3024	1.18 (0.85–1.66)	2936	1.12 (0.77–1.62)
	Breast cancer-specific mortality	3024	0.96 (0.60–1.53)	2936	0.89 (0.52–1.49)
	Non-breast cancer mortality	3024	1.58 (0.96–2.59)	2936	1.43 (0.84–2.44)
	Recurrence	2996	0.91 (0.63–1.32)	2912	0.83 (0.54–1.24)
stage I–III, only postmenopausal	Overall mortality	2755	1.15 (0.81–1.62)	2671	1.14 (0.78–1.66)
	Breast cancer-specific mortality	2755	0.89 (0.55–1.45)	2671	0.93 (0.54–1.60)
	Non-breast cancer mortality	2755	1.60 (0.97–2.63)	2671	1.44 (0.84–2.46)
	Recurrence	2729	0.88 (0.60–1.28)	2649	0.84 (0.57–1.26)

* stratified by region, adjusted for age.

** stratified by region, tumor grade, estrogen/progesterone receptor status; adjusted for age, the traditional prognostic factors tumor size, nodal status, (metastases, stage I–IV only), and for the following additional covariates evaluated using backward elimination: menopausal hormone treatment at recruitment, mode of detection, radiotherapy, and smoking. Mortality analyses are additionally adjusted for cardiovascular disease, diabetes mellitus, and body-mass index.

## Discussion

Using data from a cohort of breast cancer patients, we were not able to clearly confirm previous findings indicating an association of statin use with reduced risk of recurrence in breast cancer patients [14] and reduced breast-cancer-specific mortality [10]. Although we observed a non-significantly reduced risk of recurrence and of breast cancer-specific mortality in stage I–III breast cancer patients, our estimates of association have broad confidence intervals. This lack of precision could be due to the comparatively small number of users of lipid-lowering drugs, and due to a certain degree of misclassification of exposure, which is a main limitation of our study. Information on use of lipid-lowering drugs in general was collected in a baseline questionnaire, based on self-reported use without validation through prescription records. In addition, we did not have information on the precise type and dosage of lipid-lowering drugs. Statins are the most frequently prescribed lipid-lowering drugs (89% in Germany 2009), therefore lipid-lowering drugs could be used as a proxy for statins [1]. This assumption is supported by follow-up information from a subset of 2542 patients, providing medication use in more detail, which showed that 85% of follow-up participants taking lipid-lowering drugs actually take statins. Since statins are not sold over-the-counter but have to be prescribed by a physician for the secondary prevention of cardiovascular diseases, and are known to be usually well tolerated, it is likely that the prescription is continued after diagnosis and treatment of breast cancer. Therefore, it seems to be reasonable to use current use at diagnosis as a proxy for use after diagnosis. On the other hand, a recent study showed that the proportion of adherent statin users dropped from 64% in the year before breast cancer diagnosis to 50% during the treatment period and stayed low the subsequent three years [15]. If the adherence would also have dropped in our study, this would have led to an overestimation of the exposure. The direction of the potential bias is difficult to estimate, since we do not know if adherence is related to outcome-related variables like severity of disease.

All three previously published studies on statins and breast cancer recurrence used information on statin use after diagnosis. The reported prevalence of statin use waus between 21% and 25% [8]–[10]. The prevalence in our study was 10%, which was clearly lower. All three studies included premenopausal as well as postmenopausal patients. Kwan et al. reported a non-significant decreased risk of recurrence associated with statin use in their US cohort of nearly 2000 early stage breast cancer patients (HR 0.67, 95% CI 0.39–1.13) [9]. Chae et al. found evidence for reduced disease-free mortality (HR 0.40, 95% CI 0.24–0.67) associated with post-diagnostic statin use in a US cohort of 700 breast cancer patients diagnosed with stage II/III. Ahern et al. analyzed data from more than 18000 stage I–III breast cancer patients from Denmark, and found a beneficial effect of statin use on 5-year and 10-year-recurrence risk (HR 0.83, 95% CI 0.70–0.98) [7]. The effect was even stronger when restricting the analyses to lipophilic statins and to the lipophilic substance simvastatin. In both the Danish study and our study population, simvastatin was the most frequently used substance of all statins (71% and 87%, respectively).

Three published studies also focused their analyses on recurrence, most likely due to confounding issues with co-morbidities like cardiovascular disease, diabetes mellitus and obesity, which are strongly related to both exposure of interest and outcome. Chae et al. included overall survival as a secondary endpoint and reported no benefit (median survival 116 vs. 99 months, log-rank test p = 0.30) [8]. Our data suggest an increased overall mortality associated with use of lipid-lowering drugs due to non-breast cancer mortality. The results were not statistically significant and may be subject to residual confounding. Nielsen et al. assessed overall and cancer-related mortality in a cohort comprising all Danish cancer patients diagnosed between 1995 and 2007. For the subgroup of 46562 breast cancer patients, they reported a reduced breast-cancer specific mortality associated with statin use (HR 0.87, 95% CI 0.79–0.99) [10]. In stage I–III patients, our point estimate suggests a potential beneficial effect for breast cancer-specific mortality, which is comparable with the results of Nielsen et al. Since we censored events other than the event of interest in the analysis, it is unlikely that our results for breast cancer-specific mortality and for recurrence are severely biased by the influence of competing risks. We also have to take into account the possibility that not lipid-lowering drugs but other patient characteristics associated with the medication could be the underlying cause for the observed associations. Increased levels of cholesterol might be associated with a reduced risk of metastases [16]. On the other hand, there is evidence that increased BMI is associated with a poorer prognosis after breast cancer diagnosis [17], [18]. Hypercholesterolemia and increased BMI are indications for lipid-lowering drugs, and even though we adjusted for BMI, this might not be sufficient to rule out the possibility of confounding by indication [19].

The strengths of our analyses include the use of data from a study with comprehensive information on patient and tumor characteristics and on recurrence and mortality events. The available information allowed us to adjust for established prognostic factors. One could argue that using self-reported medication usage instead of prescription records could also be regarded as an advantage: Given the known gap between prescription and actual intake of medication, especially of statins in an elderly population, self-reported use might be more realistic than prescriptions [20], [21].

Although our results are compatible with previous findings of a beneficial effect of statins on breast cancer prognosis, they do not provide clear supportive evidence for an association with lipid-lowering drug use due to imprecise estimates.
